# Symptomatic vs. non-symptomatic device-related thrombus after LAAC: a sub-analysis from the multicenter EUROC-DRT registry

**DOI:** 10.1007/s00392-023-02237-w

**Published:** 2023-06-09

**Authors:** Vivian Vij, Ignacio Cruz-González, Roberto Galea, Kerstin Piayda, Dominik Nelles, Lara Vogt, Steffen Gloekler, Monika Fürholz, Bernhard Meier, Lorenz Räber, Gilles O’Hara, Dabit Arzamendi, Victor Agudelo, Lluis Asmarats, Xavier Freixa, Eduardo Flores-Umanzor, Ole De Backer, Lars Sondergaard, Luis Nombela-Franco, Angela McInerney, Pablo Salinas, Kasper Korsholm, Jens Erik Nielsen-Kudsk, Shazia Afzal, Tobias Zeus, Felix Operhalski, Boris Schmidt, Gilles Montalescot, Paul Guedeney, Xavier Iriart, Noelie Miton, Jacqueline Saw, Thomas Gilhofer, Laurent Fauchier, Egzon Veliqi, Felix Meincke, Nils Petri, Peter Nordbeck, Rocio Gonzalez-Ferreiro, Deepak L. Bhatt, Alessandra Laricchia, Antonio Mangieri, Heyder Omran, Jan Wilko Schrickel, Josep Rodes-Cabau, Georg Nickenig, Horst Sievert, Alexander Sedaghat

**Affiliations:** 1 Department of Cardiology, University Hospital Bonn, Venusberg-Campus 1, 53127 Bonn, Germany; 2 University Hospital of Salamanca, CIBER CV, IBSAL, Salamanca, Spain; 3 University Hospital Bern, Bern, Switzerland; 4 CardioVasculäres Centrum, Frankfurt, Germany; 5 Quebec Heart and Lung Institute, Laval University, Quebec City, Canada; 6 Hospital de la Santa Creu i Sant Pau, Barcelona, Spain; 7 Hospital Clinic Barcelona, Barcelona, Spain; 8 Rigshospitalet, University Hospital Copenhagen, Copenhagen, Denmark; 9 Hospital Clinico San Carlos Madrid, Madrid, Spain; 10 Aarhus University Hospital, Aarhus, Denmark; 11 University Hospital Düsseldorf, Düsseldorf, Germany; 12 Agaplesion Bethanien Krankenhaus, CBB, Frankfurt, Germany; 13 ACTION Study Group, Pitié-Salpêtrière Hospital (AP-HP), Sorbonne University, Paris, France; 14 University Hospital Bordeaux, Bordeaux, France; 15 Vancouver General Hospital, Vancouver, Canada; 16 University Hospital Zurich, Zurich, Switzerland; 17 University Hospital Tours, Tours, France; 18 St. Georg Hospital Hamburg, Hamburg, Germany; 19 University Hospital Würzburg, Würzburg, Germany; 20 Mount Sinai Heart, Mount Sinai Hospital, New York, USA; 21 Department of Biomedical Sciences, Humanitas University, Pieve Emanuele, Italy; 22 Humanitas Research Hospital IRCCS, Rozzano, Italy; 23 Marienkrankenhaus, Bonn, Germany; 24 Rhein-Ahr-Cardio, Bad Neuenahr-Ahrweiler, Germany

**Keywords:** Left atrial appendage closure, Atrial fibrillation, Device-related thrombus, Stroke

## Abstract

**Background:**

Device-related thrombus (DRT) after left atrial appendage closure (LAAC) is associated with adverse outcomes, i.e. ischemic stroke or systemic embolism (SE). Data on predictors of stroke/SE in the context of DRT are limited.

**Aims:**

This study aimed to identify predisposing factors for stroke/SE in DRT patients. In addition, the temporal connection of stroke/SE to DRT diagnosis was analyzed.

**Methods:**

The EUROC-DRT registry included 176 patients, in whom DRT after LAAC were diagnosed. Patients with symptomatic DRT, defined as stroke/SE in the context of DRT diagnosis, were compared against patients with non-symptomatic DRT. Baseline characteristics, anti-thrombotic regimens, device position, and timing of stroke/SE were compared.

**Results:**

Stroke/SE occurred in 25/176 (14.2%) patients diagnosed with DRT (symptomatic DRT). Stroke/SE occurred after a median of 198 days (IQR 37–558) after LAAC. In 45.8% stroke/SE occurred within one month before/after DRT diagnosis (DRT-related stroke). Patients with symptomatic DRT had lower left ventricular ejection fractions (50.0 ± 9.1% vs. 54.2 ± 11.0%, *p* = 0.03) and higher rates of non-paroxysmal atrial fibrillation (84.0% vs. 64.9%, *p* = 0.06). Other baseline parameters and device positions were not different. Most ischemic events occurred among patients with single antiplatelet therapy (50%), however, stroke/SE was also observed under dual antiplatelet therapy (25%) or oral anticoagulation (20%).

**Conclusion:**

Stroke/SE are documented in 14.2% and occur both in close temporal relation to the DRT finding and chronologically independently therefrom. Identification of risk factors remains cumbersome, putting all DRT patients at substantial risk for stroke/SE. Further studies are necessary to minimize the risk of DRT and ischemic events.

**Graphical Abstract:**

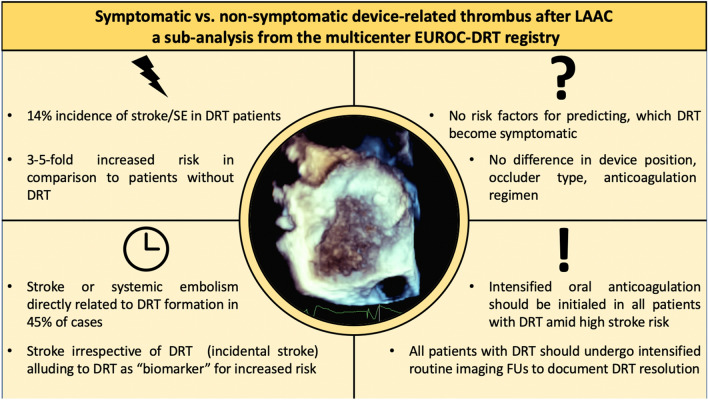

**Supplementary Information:**

The online version contains supplementary material available at 10.1007/s00392-023-02237-w.

## Background

Left atrial appendage closure (LAAC) is an established strategy for stroke prevention in patients with atrial fibrillation (AF) and contraindications against the standard treatment with oral anticoagulation (OAC) [1, 2]. Formation of device-related thrombus (DRT) has increasingly been considered as a relevant finding after LAAC and appears to be associated with impaired outcomes including increased rates of ischemic stroke and systemic embolism (SE) [3–6]. Previous studies found DRT to be related to multiple factors including patient and procedural characteristics (i.e. device position) as well as postprocedural antithrombotic regimen [4, 6–8]. Nonetheless, further data on DRT and its impact on ischemic events are warranted. In this matter it remains unclear whether DRT is directly causative for ischemic stroke or systemic embolism (SE) or rather a marker of increased thrombotic risk [7]. Also, little is known about the characteristics of stroke/SE in patients with DRT, such as the temporal correlation of the adverse event and the diagnosis of DRT as well as the LAAC procedure itself, respectively.

Therefore, this study sought to compare patients with symptomatic DRT, i.e. occurrence of stroke/SE in patients with DRT after LAAC, against patients with non-symptomatic DRT to assess stroke/SE risk in DRT patients.

## Methods

### Study population

The multicenter EUROC-DRT registry included a total of 176 patients, in whom DRT after LAAC was diagnosed during clinical follow-up (FU). Definition of DRT as used in this study has been described elsewhere [9]. In accordance with each participating center`s protocol, patients underwent regular clinical FUs after LAAC. In the case of DRT detection, patients were included in the registry. Informed consent was mandatory for all patients in each of the participating centers` registries, which were approved by the local ethics committees. All included patients received long-term clinical FU or telephone interviews to monitor the outcome. The group of patients with documented stroke/SE (including transient ischemic attack [TIA]) before or after the diagnosis of DRT was referred to as “symptomatic DRT” and compared with “non-symptomatic DRT”, meaning the group of patients with DRT but without stroke/SE. For additional analysis, patients with “symptomatic DRT” were further analyzed according to the temporal association of the thromboembolic event and the time of DRT diagnosis. Patients with stroke/SE occurring within a timeframe of one month before/after DRT diagnosis (as previously established [5]) were labeled “DRT-related stroke/SE” and compared with patients suffering a stroke/SE but beyond the given timeframe, labeled “incidental stroke/SE”. To assess risk factors for stroke/SE in patients with DRT, baseline characteristics, laboratory and echocardiographic parameters, postprocedural anticoagulation, device position and timing of stroke were compared between both groups and between DRT-related stroke/SE and all other patients.

### Echocardiographic assessment

Risk factor analysis included echocardiographic parameters as well as device position after LAAC. For this matter, the assessment included baseline transthoracic echocardiography (TTE) and transesophageal echocardiography (TEE) with an evaluation of left ventricular ejection fraction, presence of spontaneous echocardiographic contrast (SEC) (°I-°III), left atrial and ventricular volumes. Post-procedurally, 2-dimensional TEE focused on device position applying a standardized protocol as previously prescribed [7]. Evaluation of device position included assessment for complete occlusion (i.e., residual peri-device flow < 3 mm), implantation depth (measured towards the mitral annulus and along the left upper pulmonary vein [LUPV] ridge). Ostial position was achieved if LUPV ridge length was < 10 mm. Implanted occluders such as the AMPLATZER ACP and Amulet (Abbott Laboratories, Chicago, IL, USA), LAmbre (Lifetech Scientific, Shenzhen, China) and Ultraseal (Cardia Inc, Eagan, MN, USA) devices, featuring a proximal disc covering the LAA ostium, were categorized as pacifier occluders. Non-pacifier occluders included the WATCHMAN (Boston Scientific Inc, Marlborough, MA, USA), Wavecrest (Biosense Webster Inc, Irvine, CA, USA) and Occlutech LAA occluder (Occlutech International AB; Helsingborg, Sweden).

### Statistical analysis

Categorical variables are presented as frequencies with percentages included. *χ*^2^ analysis was performed for additional analysis. Continuous variables are presented as mean ± standard deviation. For comparison of central tendencies of two or more groups, Mann–Whitney *U* or Kruskal–Wallis analyses were performed, respectively. All statistical analyses were performed with SPSS software version 25.0.0.1 (IBM Corporation, Somers, NY). Statistical significance was assumed when the null hypothesis could be rejected at *p* < 0.05.

## Results

### Dynamics of symptomatic DRT

**Fig. 1:**
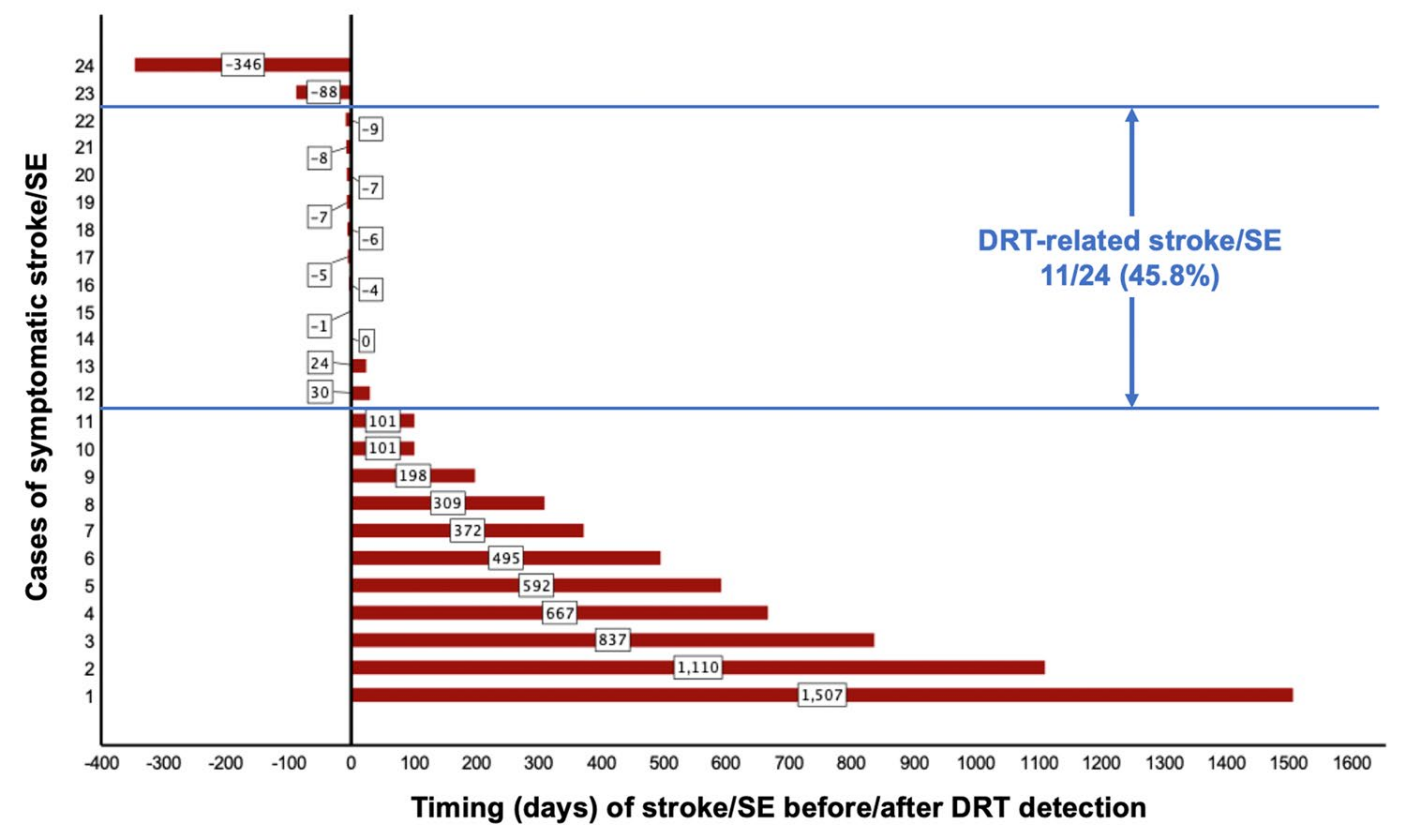
Timing of stroke/SE from DRT diagnosis in each reported case of symptomatic DRT

Out of the 176 included patients with DRT in the EUROC-DRT registry, stroke/SE occurred in 14.2% (25/176) patients. Hereby, the median maximum FU after LAAC was 682 (Interquartile range [IQR] 368–1175) days, 671 (IQR 355–1037) days in patients with symptomatic and 682 (IQR 366–1231) days in non-symptomatic DRT (*p* = 0.55). DRT were detected after a median of 93 (IQR 51–166) days after LAAC (Table 1). Exact dates of stroke/SE were available in 24/25 patients and occurred after a median of 198 (IQR 37–558) days after LAAC. In relation to DRT diagnosis, stroke/SE, therefore, occurred after a median of 27 (IQR − 7–464) days after DRT detection with 45.8% (11/24) of cases occurring within one month before/after DRT diagnosis was made (DRT-related stroke/SE) (Fig. 1). Data on anti-thrombotic regimen at the time of stroke/SE was available in 80.0% (20/25) of patients. Hereof, vitamin K antagonists (VKA) were administered in one patient (5.0%), direct oral anticoagulation (DOAC) in 4 patients (20.0%). Mainly, patients were on single antiplatelet therapy (SAPT) (*n* = 10, 50.0%) or dual antiplatelet therapy (DAPT) (*n* = 5, 25.0%). Of note, data on anti-thrombotic regimens at the time of DRT diagnosis and treatment strategies have been published elsewhere [7].

**Table 1:** Characteristics and timing of stroke/SE in patients with DRT formation (symptomatic DRT) after LAAC

	Symptomatic DRT *N* = 25
Characteristics^†^	Median (IQR) and Total (%)
Days from LAAC to the diagnosis of DRT	112 (54–366)
Days from LAAC to stroke/SE	198 (37–558)
Ischemic event within 90 days after LAAC	10 (41.7%)*
Days from diagnosis of DRT to stroke/SE	27 (− 7–464)
Within 1 month before/after DRT detection	11 (45.8%)*
Within 6 months before/after DRT detection	14 (58.3%)*
Anticoagulation at the timing of stroke/SE*	Total (%)
VKA	1 (5.0%)
DOAC	4 (20.0%)
SAPT	10 (50.0%)
DAPT	5 (25.0%)

*DAPT* dual antiplatelet therapy, *DOAC* direct oral anticoagulation, *DRT* device-related thrombosis, *IQR* interquartile range, *LAAC* left atrial appendage closure, *SAPT* single antiplatelet therapy, *SE* systemic embolism, *VKA* vitamin K antagonist

^**†**^Displayed as median with interquartile range (IQR)

*Data available in 24/25 patients only

**Fig. 2:**
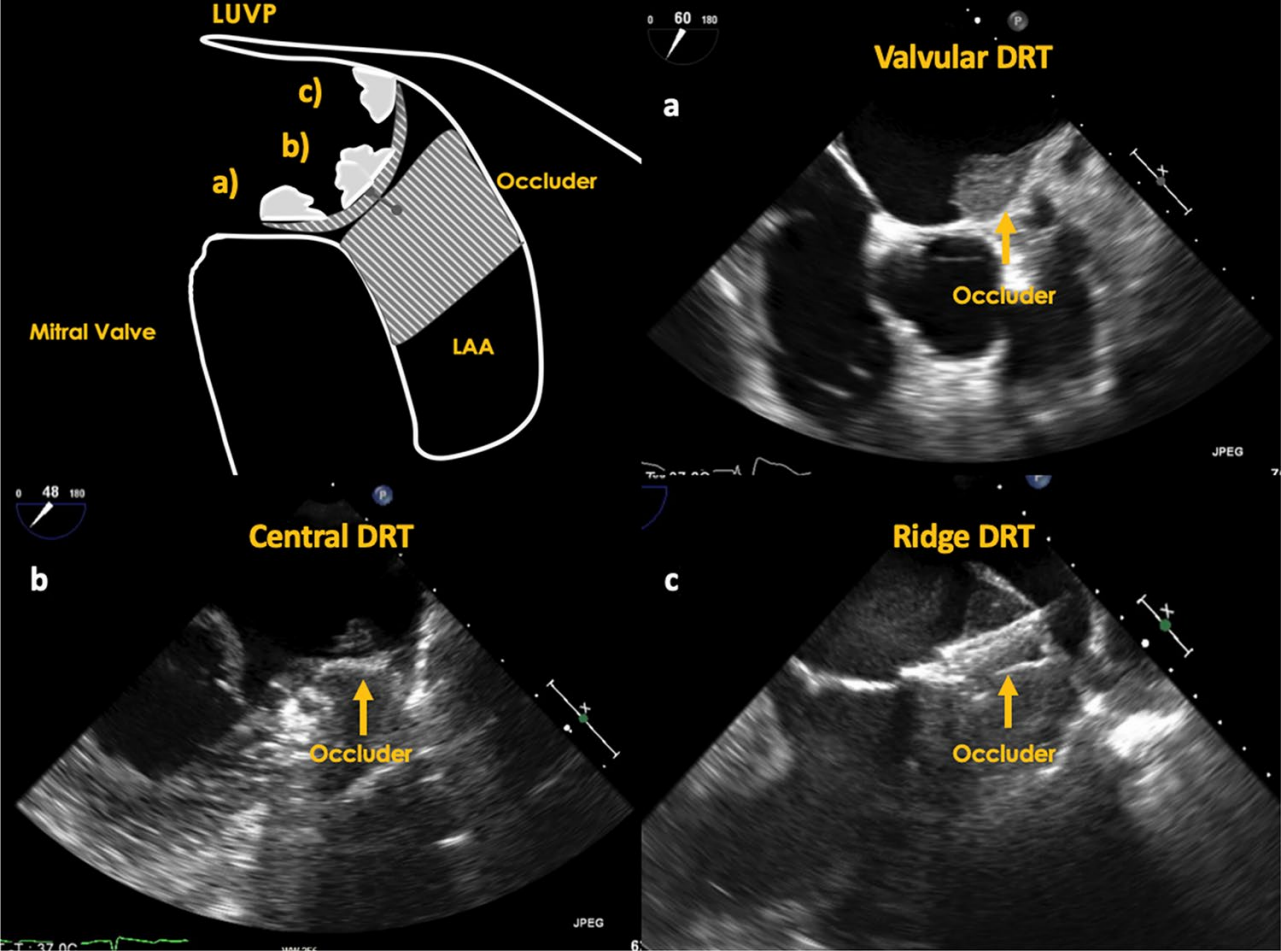
Position of DRT on LAAC occluder, **a** valvular DRT position; **b** DRT in a central occluder position, a.e. attached to screw, **c** DRT located on the ridge side of occluder in a “cul-de-sac” between LUPV and occluder

With regards to DRT, characteristics and its impact on stroke/SE, DRT were mainly located centrally on the occluder (47.3%) or along the LUPV ridge transition zone (41.9%) (Fig. 2). The remaining DRT were found on the occluder at the mitral valve side (10.9%). No difference was seen between symptomatic and non-symptomatic patients in regard to DRT position on the occluder (*p* = 0.64). DRT size measured vertically and horizontally were numerically larger in symptomatic DRT patients but missed significance (*p* = 0.22 and = 0.51, respectively).

### Baseline characteristics

Patients with symptomatic DRT were younger (73.9 ± 8.2 vs. 76.4 ± 8.4 years, *p* = 0.15) and trended to be rather male (80.0% vs. 62.9%, *p* = 0.10) than patients with non-symptomatic DRT (Table 2). Non-paroxysmal AF was more common than paroxysmal AF in the overall group of patients suffering from DRT (67.6%). This finding was even more pronounced in symptomatic DRT, with non-paroxysmal AF being present in 84.0% (21/25) and paroxysmal AF in 16.0% (4/25) of patients in this group (*p* = 0.06). Additional characteristics potentially attributing to an increased stroke risk, such as arterial hypertension (96.0% vs. 83.4%, *p* = 0.10), diabetes mellitus (36.0% vs. 21.9%, *p* = 0.12) and previous stroke/TIA (64.0% vs. 47.0%, *p* = 0.12) were documented more often in patients with symptomatic DRT. Of note, established risk scores showed no difference between patients with symptomatic and non-symptomatic DRT.

**Table 2:** Comparison of symptomatic DRT against non-symptomatic DRT

	Overall *N* = 176	Symptomatic DRT *N* = 25	Non-symptomatic DRT *N* = 151	*p* value
Max. Follow-Up after LAAC (days)	865 ± 638	783 ± 611	881 ± 645	0.55
Baseline characteristics				
Age (years)	76.0 ± 8.4	73.9 ± 8.2	76.4 ± 8.4	0.15
Male	115 (65.3%)	20 (80.0%)	95 (62.9%)	0.10
Paroxysmal AF	57 (32.4%)	4 (16.0%)	53 (35.1%)	0.06
Non-paroxysmal AF	119 (67.6%)	21 (84.0%)	98 (64.9%)	
Arterial hypertension	150 (85.2%)	24 (96.0%)	126 (83.4%)	0.10
Diabetes mellitus	42 (23.9%)	9 (36.0%)	33 (21.9%)	0.12
Prior stroke/TIA	87 (49.4%)	16 (64.0%)	71(47.0%)	0.12
HAS-BLED-Score	3.3 ± 1.2	3.4 ± 1.3	3.3 ± 1.1	0.41
ATRIA-Score	7.7 ± 2.2	7.6 ± 2.5	7.7 ± 2.2	0.59
R2CHADS2-Score	3.6 ± 1.7	4.0 ± 1.9	3.5 ± 1.7	0.27
CHADS2-Score	2.9 ± 1.3	3.2 ± 1.4	2.8 ± 1.2	0.14
CHA_2_DS_2_-VASC-Score	4.4 ± 1.8	4.6 ± 2.0	4.3 ± 1.7	0.47
GFR (ml/min/1.73m^2^)	60.4 ± 23.6	67.4 ± 22.5	59.1 ± 23.7	0.16
Echocardiographic parameters				
Left ventricular ejection fraction (%)	53.6 ± 10.8	50.0 ± 9.1	54.2 ± 11.0	0.03
LV volume diastolic (ml)	94.5 ± 35.3	110.0 ± 32.3	92.2 ± 35.4	0.15
LV volume systolic (ml)	46.4 ± 24.3	57.8 ± 23.6	44.6 ± 24.1	0.10
LA volume diastolic (ml)	92.7 ± 54.4	104.3 ± 20.1	91.0 ± 57.9	0.20
E/E’ ratio	13.5 ± 7.1	13.1 ± 6.5	13.6 ± 7.2	0.99
SEC (I-III°)	49 (45.8%)	6 (40.0%)	43 (46.7%)	0.63
Occluder and position				
Pacifier occluder	111 (63.1%)	15 (13.5%)	96 (86.5%)	0.73
Non-pacifier occluder	65 (36.9%)	10 (15.4%)	54 (84.6%)	
Occluder size (mm)	25.3 ± 3.8	25.2 ± 3.2	25.4 ± 3.9	0.93
Complete occlusion*	149 (85.6%)	20 (80.0%)	129 (86.6%)	0.39
Ostial position (LUPV ≤ 10 mm)	35 (35.0%)	4 (28.6%)	31 (36.0%)	0.59
LUPV ridge length (mm)	12.1 ± 8.5	13.6 ± 8.2	11.8 ± 8.6	0.40
Implant depth towards mitral annulus (mm)	3.3 ± 3.9	2.9 ± 3.5	3.4 ± 4.0	0.71
DRT characteristics				
Size vertically (mm)	11.2 ± 6.8	13.0 ± 7.5	10.9 ± 6.7	0.22
Size horizontally (mm)	13.2 ± 12.1	14.9 ± 16.2	12.9 ± 11.3	0.51
Position on occluder				
Valvular	14 (10.9%)	1 (5.6%)	13 (11.7%)	0.64
Central	61 (47.3%)	8 (44.4%)	53 (47.7%)	
Ridge	54 (41.9%)	9 (50.0%)	45 (40.5%)	

*AF* atrial fibrillation, *DRT* device-related thrombosis, *GFR* glomerular filtration rate, *LA* left atrium, *LAA* left atrial appendage, *LAAC* left atrial appendage closure, *LUPV* left upper pulmonary vein, *LV* left ventricle, *SE* systemic embolism, *SEC* spontaneous echocardiographic contrast, *TIA* transient ischemic attack

*Complete occlusion is defined as residual peridevice flow < 3 mm

In baseline echocardiography, patients with symptomatic DRT featured a significantly lower ejection fraction compared to patients with non-symptomatic DRT (50.0 ± 9.1% vs. 54.2 ± 11.0%, *p* = 0.03). Further echocardiographic assessment including atrial and ventricular volumes showed no significant differences.

### Occluder type and position

**Fig. 3:**
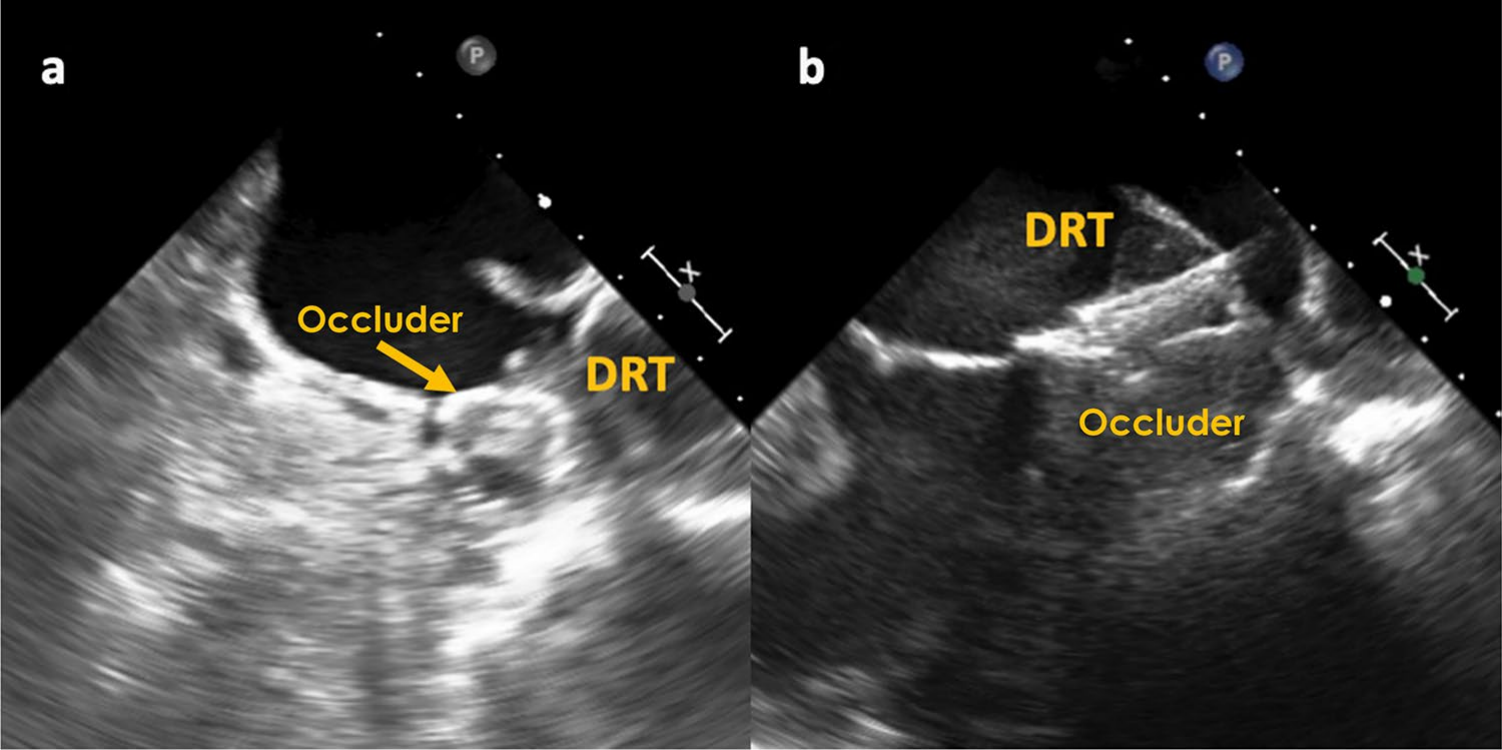
Evidence of DRT in different LAA occluder types. **a** DRT on a Watchman occluder (non-pacifier occuder), **b** DRT on an Amplatzer Amulet (pacifier occluder)

In total, pacifier occluders were implanted in 63.1% (111/176) and non-pacifier occluders in 36.9% (65/176) of patients with DRT after LAAC. Symptomatic DRT were registered equally frequently with both occluder types, with 13.5% (15/111) in pacifier occluders and with 15.4% (10/65) in non-pacifier occluders (*p* = 0.73) (Fig. 3). Further information on implanted occluders are given in Supplemental Table I. Amplatzer (58.0%) and Watchman occluder (33.5%) were mainly implanted and were accountable for all documented DRT. Other occluders were only implanted in a few cases, therefore, no DRT were detected in these patients. The rate of complete occlusion of the LAA (defined as residual peridevice flow < 3 mm) was overall satisfying (85.6%) and did not differ between both groups either (*p* = 0.39). In the overall collective, an ostial coverage of the LAA ostium was achieved roughly in a third of patients, while in patients with symptomatic DRT this rate was numerically lower than in patients with non-symptomatic DRT without achieving statistical significance (28.6% vs. 36.0%, *p* = 0.59). In this matter, no difference was seen concerning the implantation depth along the LUPV (*p* = 0.40) or along the mitral side of the LAA (*p* = 0.71).

### DRT-related stroke/SE vs. incidental stroke/SE

Symptomatic DRT were further distinguished into the temporally connected occurrence of stroke/SE (within one month before/after DRT diagnosis = DRT-related stroke) and into incidental stroke/SE (beyond one month before/after DRT diagnosis) (Supplemental Table II). Patients with temporally connected stroke/SE trended to feature an overall decreased risk for stroke compared to patients with incidental stroke/SE: Patients were numerically younger (72.3 ± 9.7 vs. 75.8 ± 6.6 years, *p* = 0.39), had lower incidences of diabetes mellitus (27.3% ± 46.2%, *p* = 0.34) and prior strokes (45.5% ± 76.9%, *p* = 0.11). In addition, established risk scores, such as the ATRIA and CHA_2_DS_2_-VASC-Score, were unanimously lower in patients with temporally connected stroke/SE. In non-pacifier occluders, DRT-related stroke/SE occurred numerically more often than incidental stroke/SE while in pacifier occluders, stroke/SE occurred more likely after a timespan of one month before/after DRT diagnosis (incidental). Additional analysis compared DRT-related stroke/SE with incidental stroke/SE and non-symptomatic DRT (Supplemental Table III), which found no significant differences.

## Discussion

Device-related thrombosis has been increasingly recognized as a relevant complication after LAAC and is linked to an increased rate of adverse events such as stroke or systemic embolism. Although the mechanism and risk factors of DRT have been described before, the relevance of DRT and its implications on patients’ outcome as well as potential treatment regimen remain poorly understood. In this matter, it remains of interest to understand DRT dynamics and its behavior to become symptomatic, i.e. cause stroke/SE.

In this study, stroke or systemic embolism occurred in approximately 14% of patients, in whom DRT were documented at one point after LAAC. This result confirms previously published studies by Alkhouli et al. [10] and Simard et al. [6], which found increased rates of 13.2% and 16.9%, respectively. Similar rates were also observed in the initial PROTECT-AF study, which described stroke in patients with DRT in 15% of cases (3/20) [2]. These findings clearly exceed the rates of stroke in patients without DRT after LAAC, which were found to be 3.8% in our own EUROC-DRT registry [11] and 3.6% in the study by Simard et al. Given the incidence of DRT after LAAC, which ranges between 3 and 4% [2, 3, 5, 6], stroke/SE after DRT presents a numerically relevant finding. Notwithstanding, detection rates of DRT are likely underestimated, as imaging follow-ups are not routinely conducted in all patients and depend on each center’s individual protocol. As previously shown, late DRT occurs in a relevant portion of patients [7], however, imaging FUs are mainly conducted within the first months after LAAC. Therefore, the rate of DRT-associated stroke/SE could also be underestimated.

Out of all symptomatic DRT in this study, stroke/SE became apparent before DRT diagnosis in approximately 45%, hereof approximately 80% within ten days before DRT diagnosis. It is likely that the embolic event initiated further imaging diagnostics, which then detected DRT as a possible cause of stroke. A temporal relation (stroke/SE within one month before/after DRT diagnosis) was seen in 45% of cases, which supports the results by Dukkipati et al. [5]. Additionally, in 42% of cases, stroke/SE occurred within a time period of 90 days after LAAC (hereof most DRT were diagnosed shortly afterwards), which is considered to be prone to DRT formation, as endocardialization of the implanted occluder surface is still incomplete [12]. This temporal relation provides support to the thesis that DRT may be directly causative of DRT, as thrombogenic formation could potentially (partially) embolize and become symptomatic.

Interestingly, and in contrast to the just given argumentation, stroke/SE occurred independently from DRT diagnosis (> 6 months before/after DRT diagnosis) in about 40% of cases (10/24). These “time-staggered” cases of symptomatic DRT may support a fundamentally opposite understanding that DRT are not directly causative but rather present a “marker”, hinting at an overall increased thrombogenic state of the patient.

This study also aimed to evaluate how symptomatic DRT differ from non-symptomatic DRT. While established risk factors for stroke/SE, such as older age, the incidence of arterial hypertension, diabetes mellitus, non-paroxysmal AF and history of stroke/TIA trended to be increased in patients with symptomatic DRT, only baseline left ventricular ejection fraction (*p* = 0.03) appeared to be predictive in univariate analysis. Of interest, device position, which has been addressed and identified as a relevant predictor for DRT formation [6, 11], did not influence the incidence of thromboembolic events in these DRT patients. Furthermore, the position of the DRT on the occluder surface as well as its size had no predictive value in our analysis. In our study, stroke/SE occurred similarly often in the pacifier and non-pacifier occluders. However, higher rates of DRT have been described in the non-pacifier occluder Watchman [13] compared to pacifier occluders [14–16]. In line with the higher rate of DRT, the incidence of ischemic events has been described to be non-inferior in pacifier occluders compared to non-pacifier occluders [17]. This corroborates randomized comparisons, documenting a higher closure rate with pacifier concluders (not found in our data) as a possible reason [17, 18]. Concluding from these findings, based on patient and procedural characteristics, it appears difficult to predict, which DRT become symptomatic and which remain non-symptomatic. This however imposes an issue of uncertainty, as no adequate consensus on DRT management and standardized treatment regimen exists. Therefore, intensified echocardiographic follow-ups and initiation of medical treatment should be considered in all patients with proof of DRT. Therefore, we advise to conduct follow-up TEE in all patients after three and six months after LAAC during the phase of endothelialisation. Depending on the risk for DRT formation, further TEE follow-ups should be routinely conducted, as late DRT are also observed. In the case of DRT diagnosis, TEE follow-ups should be intensified until DRT resolution is achieved. However, to rule out the reformation of DRT, further TEE follow-ups and modification of therapy should be considered.

As previously shown [6, 7], re-initiation of intensified antithrombotic treatment results in satisfying rates of DRT resolution, therefore the risk of stroke/SE from DRT should be carefully weighed against the risk of bleeding or intracranial hemorrhage [19]. Given the broad spectrum of available treatment regimen physicians should be encouraged to attempt medical treatment for DRT resolution. In addition, the optimal preventive post-LAAC antithrombotic treatment remains to be defined. As most centers start DAPT for 3–6 months after LAAC and eventually switch to single antiplatelet therapy, novel approaches, such as low-dose-DOAC may prove to be a feasible option. In the randomized ADRIFT study, low-dose rivaroxaban was superior to dual antiplatelet therapy to control thrombin generation while few DRT were observed only in the DAPT group [20]. Also, Cepas-Guillen et al. were able to demonstrate a superior outcome of long-term-low-dose Apixaban (2.5 mg b.i.d) treatment with reduced risk of bleeding and a combined endpoint of stroke/SE/DRT in comparison to SAPT and DAPT [21]

In summary, derived from the findings above and complementing studies, stroke or systemic embolism is a common complication in patients with a 3–fivefold increased risk in comparison to patients without DRT after LAAC. Timing of stroke/SE suggests a potential link to the formation of DRT, as stroke/SE trend to occur during the initial phase of occluder endocardialization and trend to feature a temporal relation to DRT diagnosis. As no risk factors for DRT becoming symptomatic can be derived from the results above, further randomized, prospective studies are warranted. Until then, as no standardized clinical implications on DRT management exist, the diagnosis of DRT should always demand attention and the evaluation of medical treatment, to prevent thromboembolic events.

## Limitations

The major limitation of this study is its retrospective character. All included patients with DRT were collected by the individual centers, which all followed the individual screening and follow-up protocols. Clinical data, echocardiographic FU and information on outcomes were not available in all patients. Of note, whether DRT were present in patients during the time of stroke/SE were not available in all patients, which could lead to a misinterpretation of the provided results. Also, data on antithrombotic medication and change to medical therapy after discharge, during stroke/SE are not documented in all patients. Assessment of device position was not conducted by a single core lab and therefore could be influenced by subjective data assessment. In addition, the clinical outcome of stroke/SE in DRT patients is unknown, although this information is crucial for understanding the clinical importance and impact of DRT-related stroke/SE.

### Supplementary Information

Below is the link to the electronic supplementary material.

Supplementary file1 (DOCX 32 KB)

## Abbreviations

AF: Atrial fibrillation
DOAC: Direct oral anticoagulation
DRT: Device-related thrombosis
DAPT: Dual antiplatelet therapy
FU: Follow-up
IQR: Interquartile range
LA: Left atrium
LAA: Left atrial appendage
LAAC: Left atrial appendage closure
LUPV: Left upper pulmonary vein
LV: Left ventricle
OAC: Oral anticoagulation
SAPT: Single antiplatelet therapy
SE: Systemic embolism
SEC: Spontaneous echocardiographic contrast
TIA: Transient ischemic attack
TTE: Transthoracic echocardiography
TEE: Transesophageal echocardiography
VKA: Vitamin K antagonist
