# Supplementary material for: Symptomatic vs. non-symptomatic device-related thrombus after LAAC: a sub-analysis from the multicenter EUROC-DRT registry
Source: Clin Res Cardiol. 2023 Jun 9;112(12):1790–9. doi: 10.1007/s00392-023-02237-w (PMC10697873; doi:10.1007/s00392-023-02237-w)
Supplement: Supplementary file 1 — Supplementary file1 (DOCX 32 KB) [file 392_2023_2237_MOESM1_ESM.docx]

SUPPLEMENTAL MATERIAL

**Supplemental Table I)** Overview of implanted occluders in all patients with DRT, distinguished into symptomatic and non-symptomatic DRT.

|  | **Overall**  **N=176** | **Symptomatic DRT**  **N=25** | **Non-symptomatic DRT**  **N=151** |
| --- | --- | --- | --- |
| **Occluder Type** |  |  |  |
| **Pacifier Occluder** | **111 (63.1%)** | **15 (13.5%)** | **96 (86.5%)** |
| Amplatzer Amulet | 102 (58.0%) | 15 (14.7%) | 87 (85.3%) |
| Lambre | 4 (2.3%) | 0 (0%) | 4 (100%) |
| Ultraseal | 5 (2.8%) | 0 (0%) | 5 (100%) |
| **Non-Pacifier Occluder** | **65 (36.9%)** | **10 (15.4%)** | **54 (84.6%)** |
| Watchman | 59 (33.5%) | 10 (16.9%) | 49 (83.1%) |
| Wavecrest | 1 (0.6%) | 0 (0%) | 1 (100%) |
| Occlutech | 5 (2.8%) | 0 (0%) | 5 (100%) |
| DRT = Device-related thrombosis | | | |

**Supplemental Table II)** Comparison of patients with DRT-related stroke/SE (within one month) and incidental stroke/SE (beyond one month)

|  | **Symptomatic DRT**  **N=24^†^** | **DRT-related stroke/SE**  **N=11** | **Incidental stroke/SE**  **N=13** | **p-value** |
| --- | --- | --- | --- | --- |
| **Baseline characteristics** |  |  |  |  |
| Age (years) | 74.2±8.2 | 72.3±9.7 | 75.8±6.7 | 0.39 |
| Male | 19 (79.2%) | 7 (63.6%) | 12 (92.3%) | 0.09 |
| Paroxysmal AF | 4 (16.7%) | 3 (27.3%) | 1 (7.7%) | 0.20 |
| Non-paroxysmal AF | 20 (83.3%) | 8 (72.7%) | 12 (92.7%) |  |
| Arterial hypertension | 23 (95.8%) | 10 (90.9%) | 13 (100%) | 0.27 |
| Diabetes mellitus | 9 (37.5%) | 3 (27.3%) | 6 (46.2%) | 0.34 |
| Prior stroke/TIA | 15 (62.5%) | 5 (45.5%) | 10 (76.9%) | 0.11 |
| HAS-BLED-Score | 3.4±1.3 | 3.4±1.3 | 3.5±1.2 | 0.73 |
| ATRIA-Score | 7.6±2.6 | 7.2±2.6 | 7.9±2.6 | 0.46 |
| R2CHADS2-Score | 3.9±1.9 | 3.3±1.6 | 4.5±2.1 | 0.15 |
| CHADS2-Score | 3.3±1.5 | 2.6±1.2 | 3.9±1.4 | 0.03 |
| CHA_2_DS_2_-VASC-Score | 4.6±2.0 | 3.9±1.8 | 5.2±2.0 | 0.11 |
| GFR (ml/min/1.73m^2^) | 67.4±22.5 | 70.0±26.6 | 66.4±20.3 | 1 |
| **Echocardiographic parameters** |  |  |  |  |
| Left ventricular ejection fraction (%) | 50.0±9.1% | 50.8±7.7% | 49.4+10.3% | 0.78 |
| SEC (I-III°) | 6 (40.0%) | 2 (33.3%) | 4 (66.7%) | 0.67 |
| Pacifier occluder | 14 (58.3%) | 5 (45.5%) | 9 (69.2%) | 0.24 |
| Non-pacifier occluder | 10 (15.4%) | 6 (54.5%) | 4 (30.8%) |  |
| Occluder size (mm) | 25.3±3.3 | 25.5±4.2 | 25.0±2.3 | 0.78 |
| Complete occlusion* | 19 (79.2%) | 10 (90.9%) | 9 (69.2%) | 0.19 |
| Ostial position (LUPV ≤10 mm) | 4 (28.6%) | 3 (50.0%) | 7 (85.7%) | 0.12 |
| LUPV ridge length (mm) | 13.6±8.2 | 12.4±8.9 | 14.4±8.2 | 0.62 |
| Implant depth towards mitral annulus (mm) | 2.9±3.5 | 4.2±4.5 | 2.1±2.8 | 0.28 |
| DRT Size vertically (mm) | 13.0±7.5 | 13.3±9.6 | 12.8±6.1 | 0.97 |
| DRT Size horizontally (mm) | 14.9±16.2 | 9.3±5.1 | 18.9±20.1 | 0.19 |
| AF = Atrial fibrillation, DRT = Device-related thrombosis, GFR = glomerular filtration rate, LAA = Left atrial appendage, LUPV = Left upper pulmonary vein, LV = Left ventricle, SEC = Spontaneous echocardiographic contrast, TIA = Transient ischemic attack,  ^†^ Timing of stroke/SE and further information documented in 24/25 cases  * complete occlusion is defined as residual peridevice flow <3 mm | | | | |

**Supplemental Table III)** Comparison of patients with DRT-related stroke/SE (within one month) and patients with incidental stroke/SE or non-symptomatic DRT

|  | **Overall**  **N=176** | **DRT related stroke/SE**  **N=11** | **Incidental stroke/SE & non-symptomatic DRT**  **N=165** | **p-value** |
| --- | --- | --- | --- | --- |
| **Baseline characteristics** |  |  |  |  |
| Age (years) | 76.0±8.4 | 72.3±9.7 | 76.2±8.3 | 0.19 |
| Male | 115 (65.3%) | 7 (63.6%) | 108 (65.5%) | 0.90 |
| Paroxysmal AF | 57 (32.4%) | 3 (27.3%) | 54 (32.7%) | 0.71 |
| Non-paroxysmal AF | 119 (67.6%) | 8 (72.3%) | 111 (67.3%) |  |
| Arterial hypertension | 150 (85.2%) | 10 (90.9%) | 140 (84.8%) | 0.58 |
| Diabetes mellitus | 42 (23.9%) | 3 (27.3%) | 39 (23.6%) | 0.78 |
| Prior stroke/TIA | 87 (49.4%) | 5 (45.5%) | 82 (49.7%) | 0.79 |
| HAS-BLED-Score | 3.3±1.2 | 3.4±1.4 | 3.3±1.1 | 0.74 |
| ATRIA-Score | 7.7±2.2 | 7.7±2.6 | 7.7±2.2 | 0.63 |
| R2CHADS2-Score | 3.6±1.7 | 3.3±1.6 | 3.6±1.7 | 0.57 |
| CHADS2-Score | 2.9±1.3 | 2.6±1.2 | 2.9±1.3 | 0.32 |
| CHA_2_DS_2_-VASC-Score | 4.4±1.8 | 3.9±1.8 | 4.4±1.8 | 0.33 |
| GFR (ml/min/1.73m^2^) | 60.4±23.6 | 69.0±26.7 | 59.8±23.4 | 0.27 |
| **Echocardiographic parameters** |  |  |  |  |
| Left ventricular ejection fraction (%) | 53.6±10.8 | 50.8±7.7 | 52.8±11.0 | 0.23 |
| SEC (I-III°) | 49 (45.8%) | 2 (33.3%) | 47 (46.5%) | 0.53 |
| Pacifier occluder | 111 (63.1%) | 5 (4.5%) | 106 (95.5%) | 0.21 |
| Non-pacifier occluder | 65 (36.9%) | 6 (9.2%) | 59 (90.8%) |  |
| Occluder size (mm) | 25.3±3.8 | 25.5±4.2 | 25.3±3.8 | 0.80 |
| Complete occlusion* | 149 (85.6%) | 10 (90.9%) | 139 (85.3%) | 0.61 |
| Ostial position (LUPV ≤10 mm) | 35 (35.0%) | 3 (50.0%) | 32 (34.0%) | 0.48 |
| LUPV ridge length (mm) | 12.1±8.5 | 12.4±8.9 | 12.1±8.5 | 0.93 |
| Implant depth towards mitral annulus (mm) | 3.3±3.9 | 4.2±4.5 | 3.2±3.9 | 0.47 |
| DRT Size vertically (mm) | 11.2±6.8 | 13.3±9.6 | 11.1±6.6 | 0.61 |
| DRT Size horizontally (mm) | 13.2±12.1 | 9.3±5.1 | 13.4±12.3 | 0.65 |
| AF = Atrial fibrillation, DRT = Device-related thrombosis, GFR = glomerular filtration rate, LAA = Left atrial appendage, LUPV = Left upper pulmonary vein, LV = Left ventricle, SEC = Spontaneous echocardiographic contrast, TIA = Transient ischemic attack,  ^†^ Timing of stroke/SE and further information documented in 24/25 cases  * complete occlusion is defined as residual peridevice flow <3 mm | | | | |
